# A novel clinical−radiomic nomogram for the crescent status in IgA nephropathy

**DOI:** 10.3389/fendo.2023.1093452

**Published:** 2023-01-20

**Authors:** Xiachuan Qin, Linlin Xia, Xiaomin Hu, Weihan Xiao, Xian Huaming, Xie Xisheng, Chaoxue Zhang

**Affiliations:** ^1^ Department of Ultrasound, The First Affiliated Hospital of Anhui Medical University, Hefei, Anhui, China; ^2^ Department of Ultrasound, Nanchong Central Hospital, The Second Clinical Medical College, North Sichuan Medical College (University), Nanchong, Sichuan, China; ^3^ Department of Nephrology, Nanchong Central Hospital, The Second Clinical Medical College, North Sichuan Medical College (University), Nanchong, Sichuan, China

**Keywords:** IgA nephropathy, crescents, machine learning, radiomics, nomogram

## Abstract

**Objective:**

We used machine-learning (ML) models based on ultrasound radiomics to construct a nomogram for noninvasive evaluation of the crescent status in immunoglobulin A (IgA) nephropathy.

**Methods:**

Patients with IgA nephropathy diagnosed by renal biopsy (n=567) were divided into training (n=398) and test cohorts (n=169). Ultrasound radiomic features were extracted from ultrasound images. After selecting the most significant features using univariate analysis and the least absolute shrinkage and selection operator algorithm, three ML algorithms were assessed for final radiomic model establishment. Next, clinical, ultrasound radiomic, and combined clinical−radiomic models were compared for their ability to detect IgA crescents. The diagnostic performance of the three models was evaluated using receiver operating characteristic curve analysis.

**Results:**

The average area under the curve (AUC) of the three ML radiomic models was 0.762. The logistic regression model performed best, with AUC values in the training and test cohorts of 0.838 and 0.81, respectively. Among the final models, the combined model based on clinical characteristics and the Rad score showed good discrimination, with AUC values in the training and test cohorts of 0.883 and 0.862, respectively. The decision curve analysis verified the clinical practicability of the combined nomogram.

**Conclusion:**

ML classifier based on ultrasound radiomics has a potential value for noninvasive diagnosis of IgA nephropathy with or without crescents. The nomogram constructed by combining ultrasound radiomic and clinical features can provide clinicians with more comprehensive and personalized image information, which is of great significance for selecting treatment strategies.

## Introduction

Immunoglobulin A nephropathy (IgAN) is the most common primary glomerulonephritis worldwide (1). Approximately 40% of patients with IgAN will develop end-stage renal disease within 10−20 years (2). To determine the appropriate treatment for prevention of disease progression, pathological results are often required. At present, the Oxford classification is the global standardized pathological classification of IgAN, which aims to predict renal results at biopsy and during follow-up according to the MEST-C criteria (3, 4). Among these, the presence of crescents is a new indicator proposed in 2017 (4).

The formation of crescents is a common histopathological change in IgAN, which occurs in approximately 20−60% of patients. Patients with crescentic IgAN have more serious clinical and pathological findings (5, 6). In addition, the presence of crescents is usually related to rapid renal function decline and indicates an increased risk of poor renal prognosis (4, 7, 8). Thus, the crescent status is an independent predictor of IgAN progression; however, it may change over time (9–11). Importantly, in addition to its value as a marker of disease progression, it also indicates responsiveness to immunosuppressive therapy (8).

Currently, renal biopsy is the only way to confirm the crescent status in IgAN, but it is an invasive examination that may lead to bleeding, fistula formation, and other complications, and even death (12, 13). Furthermore, some patients decline undergoing renal biopsy due to fear. Therefore, although the crescent status of IgAN change over time, it is difficult to repeat renal biopsy (14).

Ultrasound is a relatively cheap and widely used imaging technique, and is used as a first-line means for renal disease examination (15, 16). However, the information obtained by sonographers with naked eyes is limited. Radiomics can extract and quantify high-throughput imaging biomarkers beyond the human perceptible range. Combining these biomarkers with various machine learning (ML) technologies allows to effectively identify subtle and complex changes in tissues (17–19). To the best of our knowledge, no studies to date have documented the use of ultrasound radiomics for noninvasive assessment of the crescent status in IgAN.

Therefore, the purpose of this study was to develop and validate a nomogram combining an ML model based on ultrasound radiomics with clinical factors for personalized noninvasive assessment of the crescent status in patients with IgAN.

## Methods

### Study design and population

The study was approved by the Institutional Review Committee of the First Affiliated Hospital of Anhui Medical University (approval number PJ2022-11-29). The requirement for informed consent was waived due to the retrospective study design and use of deidentified data.

We retrospectively reviewed the records of patients with IgAN who underwent renal biopsy at the First Affiliated Hospital of Anhui Medical University from January 2019 to May 2022. The inclusion criteria were as follows: 1) IgAN confirmed by renal puncture biopsy; and 2) more than 10 glomeruli were observed under light microscope. The following were the exclusion criteria: 1) acute renal damage and valvular heart disease; 2) renal artery stenosis or urinary tract obstruction; 3) renal cysts or tumors; and 4) Doppler ultrasound finding indicating renal artery stenosis (20).

**Figure 1:**
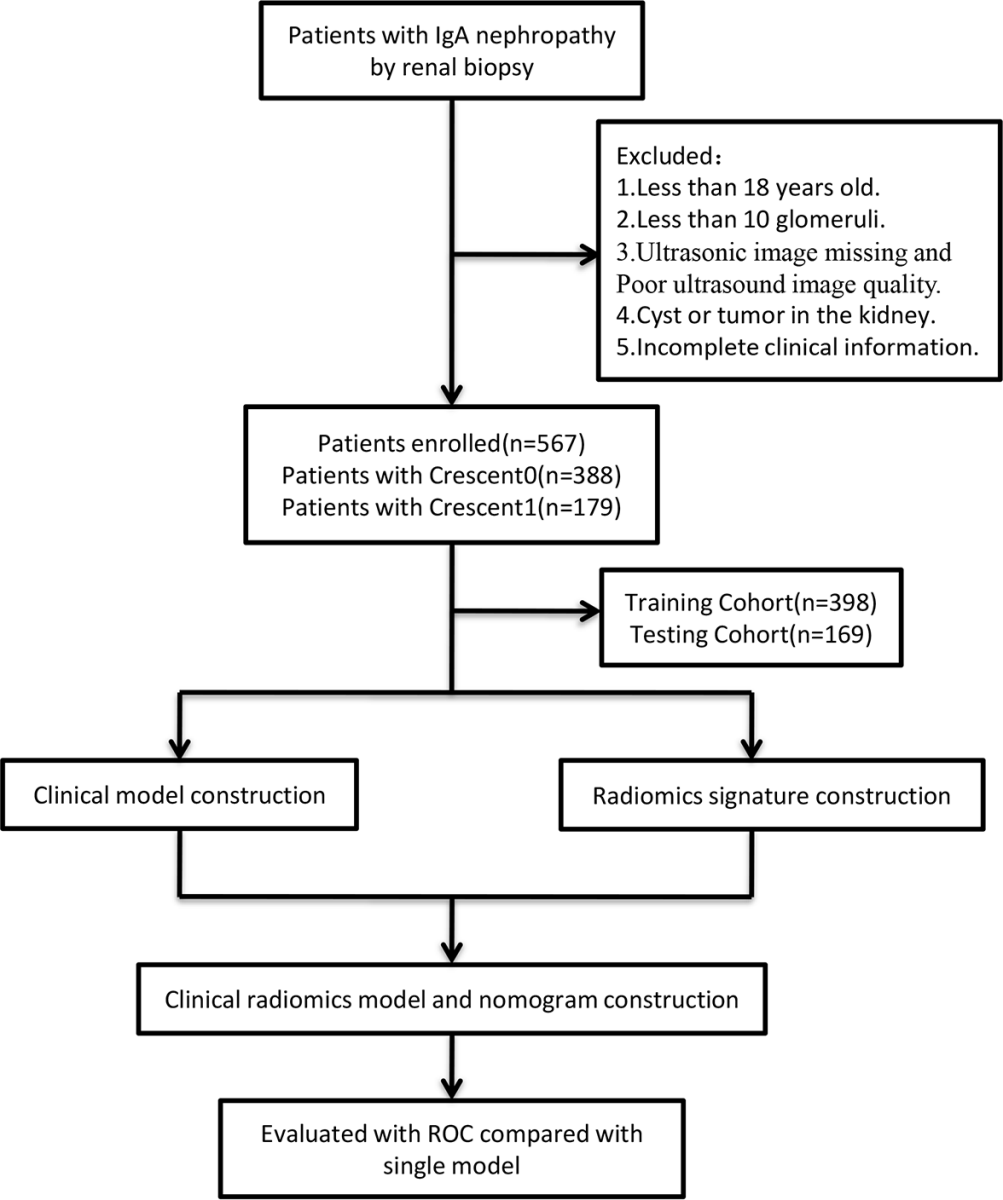
The flow diagram of the study.

The enrolled patients were randomly divided into training and test cohorts at a ratio of 7:3. The following baseline data were collected: sex, age, systolic blood pressure, diastolic blood pressure, platelet count, hemoglobin, creatinine, urea, and uric acid levels, estimated glomerular filtration rate, urine protein level, 24-h urine protein level, 24-h urine volume, presence of occult hematuria, and number of urinary red blood cells. The study flowchart is shown in **Figure 1**.

### Ultrasound examination

Renal ultrasound examination was performed by four sonographers with 5, 6, 8, and 10 years of experience in routine ultrasound examination with the Mindray Resona 7 device (Shenzhen Mindray BioMedical Electronics Co., China) and GE Vivid E9 (General Electric Co., USA), using a multifrequency (2−5 MHZ) convex array probe (C5-2).

All patients were evaluated after overnight fasting in the supine position. The ultrasound probe was gently positioned over the right abdomen in an oblique projection way to visualize the kidney as a longitudinal image and obtain a coronary ultrasound image of the right kidney in the largest cross-section. All measurements were taken during apnea at the end of inspiration. The parameter configuration during the acquisition process was based on the best display settings of ultrasound images.

### Renal biopsy

Renal biopsy was performed within 3 days after the renal ultrasound examination by two experienced nephrologists. The right kidney was selected for biopsy. The paraffin-embedded sections were stained with hematoxylin and eosin, periodate Schiff, trimethylamine silver, and Masson’s trichrome. The biopsy specimens of all patients were evaluated using immunofluorescence, light, or electron microscopy.

The pathological variables of IgAN were scored according to the MEST-C criteria: mesangial cell increase, capillary cell increase, segmental glomerulosclerosis, tubular atrophy/interstitial fibrosis, and presence of crescents. The presence of crescents was graded according to the proportion of glomeruli with cellular or fibrocellular crescents, as follows: C0, absent; C1, 0–25% of glomeruli; and C2, ≥ 25% of glomeruli (7). Due to the limited sample size, C1 and C2 cases were combined into one group.

### Clinical model construction

Univariate logistic regression analysis was used to analyze the correlation between clinical parameters and the presence of crescents. The variables with a significant correlation (*P* < 0. 05) were included in the multivariate logistic regression analysis to determine the independent predictive factors significantly related to the presence of crescents. These were used to establish a clinical model.

### Ultrasound image segmentation and radiomic feature extraction

Renal ultrasound image segmentation was performed by the reader 1 (with 9 years of abdominal ultrasound imaging experience) and the reader 2 (with 7 years of abdominal ultrasound imaging experience) using the ITK (software v3.8.0, http://www.itksnap.org/pmwiki/pmwiki.php?n=Downloads.SNAP3). Regions of interests (ROIs) were manually selected and segmented. Ultrasound radiomic feature extraction was performed using the PyRadiomics (software v3.0.1, https://github.com/AIM-Harvard/pyradiomics), which can extract lots of features from ultrasound images using a large number of engineering algorithms (**Figure 1**).

First, renal ultrasound images of 50 patients were randomly selected, and the ROIs were delineated by reader 1 and reader 2, respectively. The same procedure was repeated by reader 1 after 2 weeks with renal ultrasound images of another randomly selected 50 patients. The consistency of the extracted features for each reader (inter-class correlation coefficient) and between two readers (intra-class correlation coefficient) was tested using intra- and interclass correlation analysis, respectively. The same procedure was repeated after 2 weeks with renal ultrasound images of another randomly selected 50 patients. Intra- and interclass correlation coefficient values larger than 0.75 were considered to indicate good consistency of the extracted features. The image segmentation and radiomic feature extraction for the remaining ultrasound images was completed by reader 1 alone. Only the features with good consistency were used in subsequent analyses.

### Ultrasound radiomic model construction

**Figure 2:**
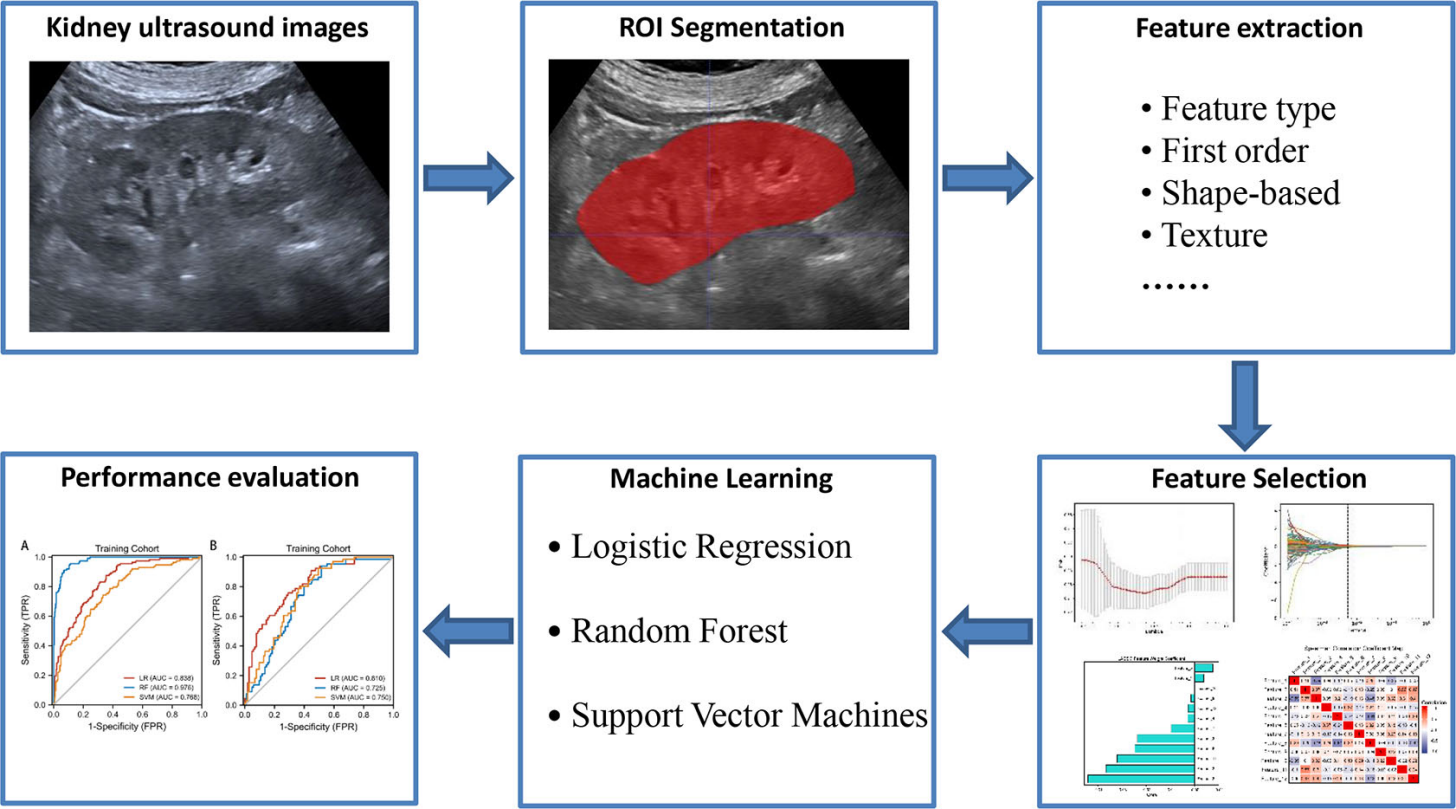
The radiomics flow chart of the study.

As shown in **Figure 2**, The radiomic features with intra- and interclass correlation coefficient values larger than 0.75 in the training cohort were included in univariate analysis to identify features with a significant distribution difference between the C0 and C1 groups in the training cohort (*P* < 0.05). The identified features were analyzed using the least absolute shrinkage and selection operator algorithm to select the most significant features for predicting the crescent status.

Next, we constructed three radiomic models that were trained with the selected features using different ML algorithms, including logistic regression (LR), random forest (RF), and support vector machines (SVM). Five-fold cross-validation was performed in the training dataset to obtain the best parameter configuration. The super parameters of the three ML algorithms were adjusted through the grid search method and five-fold cross-validation in the training dataset. In each loop of CV, the super parameters with the best area curve (AUC) under the receiver operating characteristic (ROC) value were retained, and the entire training dataset was used for the final model establishment. The remaining patients in the test dataset were used to evaluate the model performance. After completing each round of CV, each patient was assigned the prediction probability.

### Statistical analysis

Statistical analysis was performed using IBM SPSS Statistics version 25.0(IBM Corp., Armonk, NY, USA) and Python 2.2(Python Software Foundation, Beaverton, OR, USA). Quantitative data with normal distribution were expressed as mean ± standard deviation values, while those with non-normal distribution were expressed as median ± interquartile range values. Categorical data were expressed as numbers and percentages. The Chi square test, independent sample Student’s t-test, and Mann-Whitney’s U test were used for univariate analysis. The DeLong test was employed to compare the AUC values of the three models in the training and test cohorts. Two-sided *P* values of less than 0.05 were considered to indicate statistical significance.

## Results

### Patients’ clinical characteristics

A total of 567 patients with IgAN confirmed by renal biopsy met the eligibility criteria, including 279 men and 288 women with a mean age of 39.4 ± 12.3 years. Among them, 398 were assigned to the training cohort and 169 to the test cohort. The clinical characteristics of the patients in the training and test cohorts according to the crescent status are shown in **Table 1**.

**Table 1:** Clinical factors in the training and testing cohorts.

Clinical factors	Training cohort (n =398)			Testing cohort (n= 169)		
	Crescent1	Crescent0	P	Crescent1	Crescent0	P
Age (years)	39.9 ± 12.8	39.4 ± 12.2	0.733	38.8 ± 12.1	39.4 ± 12.4	0.745
Sex(male/female)	55/58	146/139	0.647	26/40	52/51	0.16
Systolic pressure (mmHg)	132.1 ± 16.4	132.1 ± 19.2	0.998	131.1 ± 18	132.3 ± 18.3	0.658
Diastolic pressure (mmHg)	87.1 ± 15	86.3 ± 12.5	0.5566	85.9 ± 12.5	87.3 ± 13.4	0.496
Creatinine level at biopsy(μmol/L)	97.9 ± 44.3	91.9 ± 49.8	0.259	96.3 ± 39.1	91.8 ± 44.9	0.5
Urine occult blood(Ery/μl)	2.76 ± 0.344	2.17 ± 1.13	0.000	2.65 ± 0.75	1.91 ± 1.2	0.000
Urinary erythrocytes(a/μl)	88.3 ± 130.3	64.3 ± 136.2	0.108	74.2 ± 142.7	48.4 ± 101.8	0.172
eGFR at biopsy(mL/min/1.73m^2^)	88.1 ± 30.9	94.1 ± 32.5	0.091	87.4 ± 30.3	94.4 ± 44.9	0.147
Hemoglobin at biopsy(g/L)	130 ± 17.8	134.1 ± 17	0.021	131.7 ± 21.8	134.6 ± 21.1	0.402
Urea at biopsy(mmol/L)	6.56 ± 3.01	6.14 ± 2.4	0.142	6.19 ± 2.27	6.2 ± 2.28	0.926
Uric acid at biopsy(μmol/L)	392.8 ± 103.9	383.5 ± 104.7	0.426	381.2 ± 98.9	377.5 ± 104.6	0.818
Platelet at biopsy(10^9^/L)	234.4 ± 65.8	234.8 ± 67.5	0.226	243.7 ± 73	233.9 ± 63	0.364
Urine protein(g/L)	1.25 ± 1.24	0.82 ± 1.43	0.041	1.1 ± 1.16	0.72 ± 0.82	0.034
24h Urine protein(g/24H)	2.18 ± 1.92	1.3 ± 1.80	0.373	1.83 ± 1.16	1.25 ± 1.44	0.054
24h urine volume(L)	1.86 ± 0.62	1.85 ± 0.64	0.907	1.81 ± 0.58	1.81 ± 0.69	0.91

### Clinical model construction

In the univariate analysis, the following clinical parameters showed statistically significant differences according to the crescent status: presence of occult hematuria (P<0.001), hemoglobin level (P=0.021) and urine protein level (P=0.041). The multivariate regression analysis showed that presence of occult hematuria was an independent predictor of the crescent status.

### Ultrasound radiomic feature extraction, selection, and model construction

A total of 1 504 radiomic features were extracted from ultrasound images. Through intra- and interclass correlation analysis and subsequent univariate correlation analysis, 236 features were found to be significantly different between the C0 and C1 groups. Among them, the least absolute shrinkage and selection operator algorithm and multivariable logistic analysis identified the following 12 features as the most significant: original_glcm_Correlation,original_glszm_HighGrayLevelZoneEmphasis, original_ngtdm_Complexity, original_ngtdm_Strength, wavelet-HLL_glcm_MCC, wavelet-HLH_gldm_SmallDependenceHighGrayLevelEmphasis, wavelet-HLH_glszm_SizeZoneNonUniformityNormalized, wavelet-HHL_glszm_SmallAreaLowGrayLevelEmphasis, wavelet-LHL_glszm_LargeAreaLowGrayLevelEmphasis,wavelet-HHH_glcm_SumAverage,square_glszm_SmallAreaHighGrayLevelEmphasis, squareroot_firstorder_Maximum. These were included in the ultrasound radiomic models.

### Diagnostic performance of the ultrasound radiomic models

**Figure 3:**
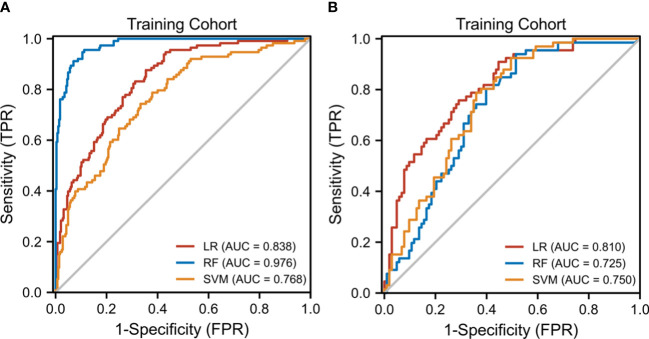
The receiver operating characteristic (ROC) curves of the three ML models. **(A)** Three ML model ROC curves in the training cohort. **(B)** Three model ML ROC curves in the testing cohort.

The diagnostic performance of the three radiomic models based on different ML algorithms is presented in **Table 2**. The ROC curves of these models in the training and test cohorts are shown in **Figure 3**. The average AUC value of the three models for determining the crescent status was 0.762. Among them, the LR model performed best, with an AUC value, accuracy, sensitivity, specificity, negative predictive value, and positive predictive value of 0.838, 71.1, 83.6%, 64.6%, 49.5%, and 92.9% in the training cohort, and 0.81, 72.8, 75.8%, 70.9%, 62.5%, and 82% in the test cohort, respectively.

**Table 2:** Performance of the three model in the training and testing cohorts.

	AUC	ACC	SEN	SPE	PPV	NPV
Training cohort						
Random Forest	0.976(0.964-0.988)	90.7	0.956	0.888	0.771	0.981
Support Vector Machines	0.768(0.717-0.818)	68.8	0.743	0.667	0.469	0.868
Logic Regression	0.838(0.797-0.878)	71.1	0.876	0.646	0.495	0.929
Testing cohort						
Random Forest	0.725(0.649-0.801)	66.3	0.939	0.485	0.539	0.926
Support Vector Machines	0.75(0.678-0.822)	66.9	0.924	0.505	0.545	0.912
Logic Regression	0.81(0.745-0.874)	72.8	0.758	0.709	0.625	0.82

AUC, area under the curve; ACC, accuracy; SEN, sensitivity; SPE, specificity; PPV, Positive likelihood ratio; NPV, negative likelihood ratio.

### Clinical−radiomic nomogram

**Figure 4:**
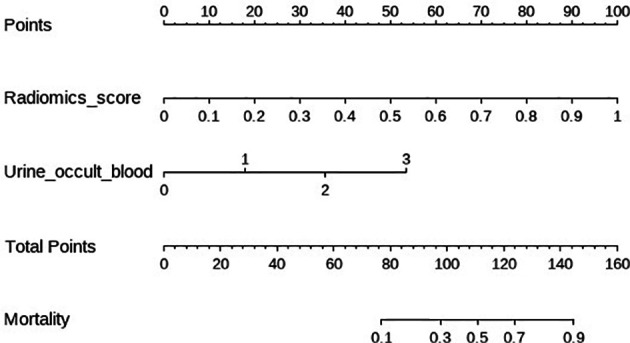
The clinical radiomics nomogram. The values of clinical characteristics and rad score can be converted into quantitative values according to the points axis. After summing the individual points to achieve the final sum shown on the total points axis, The evaluation of this crescent is shown.

**Figure 5:**
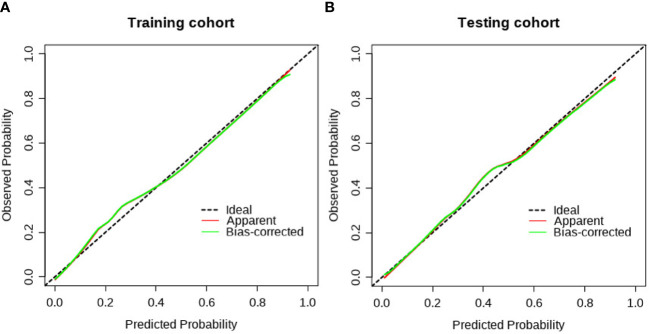
The calibration curve of the clinical radiomics model. **(A)** The calibration plot also showed good agreement between the transition probabilities predicted by the nomogram in the training cohort; **(B)** The calibration plot also showed good agreement in the testing cohort.

The clinical**−**radiomic nomogram was established by combining the Rad score and clinical characteristics (**Figure 4**). The combined model had an AUC value, accuracy, sensitivity, and specificity of 0.883, 77.6, 91.2%, and 72.3% in the training cohort, and 0.862, 78.1, 86.4%, and 72.8% in the test cohort, respectively. The calibration curve of the combined model showed good consistency between the predicted and actual crescent status in both cohorts (**Figure 5**).

### Comparison of the three diagnostic models

**Figure 6:**
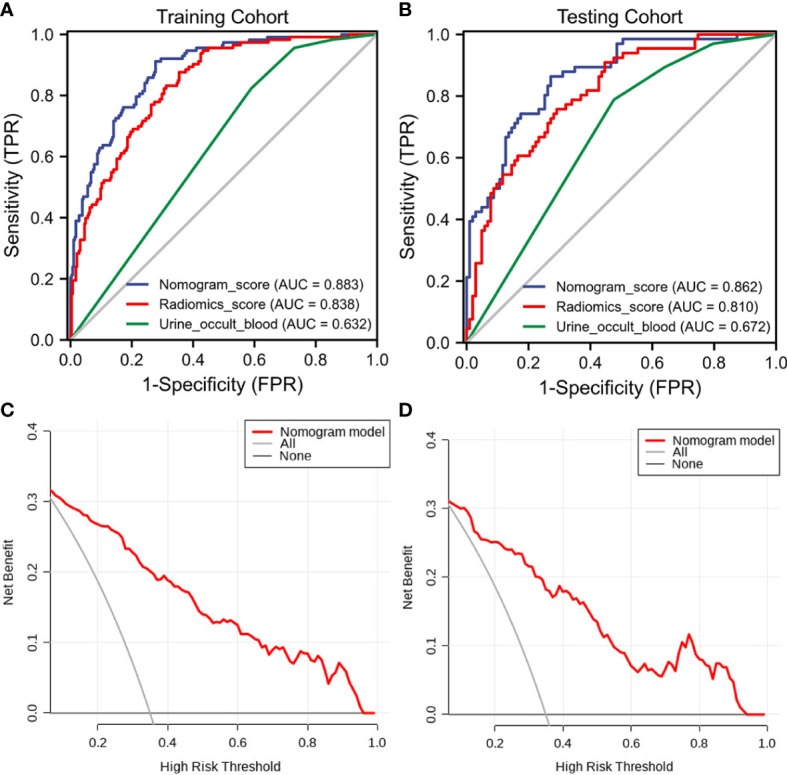
The receiver operating characteristic (ROC) curves and decision curve analysis (DCA) of the three models of the three models. **(A)** Three model ROC curves in the training cohort. **(B)** Three model ROC curves in the testing cohort. **(C)** Three DCA models in the training cohort. **(D)** Three DCA models in the testing cohort.

The discriminant effectiveness of the three diagnostic models (clinical, ultrasound radiomic, and clinical**−**radiomic) is shown in **Table 3**. The ROC curves of the three models in the training and test cohorts are shown in **Figure 6**. The decision curve analysis confirmed the clinical decision effectiveness of the combined model (**Figure 6**).

**Table 3:** Performance of the clinical model, radiomics model, and clinical radiomics model in the training and testing cohorts.

	AUC	ACC	SEN	SPE	PPV	NPV
Training cohort						
Clinical	0.632(0.589-0.675)	52.8	0.823	0.411	0.356	0.854
Radiomics	0.838(0.797-0.878)	71.1	0.876	0.646	0.495	0.929
Clinical Radiomics	0.883 (0.849-0.918)	77.6	0.912	0.723	0.566	0.954
Testing cohort						
Clinical	0.672(0.603-0.74)	62.7	0.788	0.524	0.515	0.794
Radiomics	0.81(0.745-0.874)	72.8	0.758	0.709	0.625	0.82
Clinical Radiomics	0.862 (0.807-0.917)	78.1	0.864	0.728	0.671	0.893

AUC, area under the curve; ACC, accuracy; SEN, sensitivity; SPE, specificity; PPV, Positive likelihood ratio; NPV, negative likelihood ratio.

## Discussion

In the present study, we identified 12 radiomic features from renal ultrasound images and used them to construct prediction models with various classification algorithms to determine the crescent status in IgAN. These models exhibited good performance in discriminating between presence and absence of crescents, with an average AUC value of 0.762 in the test cohort, with the LR model performing best(the AUC value was 0.81). Furthermore, we constructed a combined clinical**−**radiomic nomogram, which had AUC values as high as 0.883 and 0.862 in the training and test cohorts, respectively. These findings indicate that ML has great application potential in the field of renal ultrasound due to its powerful processing capacity for high-throughput data. However, the prediction effectiveness of the combined clinical**−**radiomic nomogram can be further improved. To the best of our knowledge, this is the first study on the application of ML model analysis of ultrasound radiomic features for noninvasive evaluation of the crescent status in IgAN.

Although ultrasound is the first-line imaging method for renal examination at present, subtle changes are more difficult to identify using this method. As this tissue heterogeneity is beyond human perception, it can be analyzed by obtaining non-visual information using mathematical formulas to extract quantitative texture features through ultrasound radiomics (21, 22). Compared with the traditional morphological features, radiomic features may provide more comprehensive and quantitative information on renal heterogeneity, and help to explain the potential relationship between pathophysiological properties and radiographic imaging phenotypes.

As a branch of artificial intelligence, ML can perform classification by building an algorithm model, and improve its performance based on some experience (data) (23, 24). In recent years, some studies have attempted to diagnose chronic kidney disease (CKD) by ultrasound radiomics, showing great potential. Bandara et al. performed two-dimensional ultrasound on a group of patients with CKD (n = 75) and healthy subjects (n = 27), and found that the radiomic features based on wavelet transform were sensitive to the directivity of ultrasound speckle patterns, and could be successfully used to distinguish CKD and healthy kidney ultrasound images (25). Kim et al. set three ROIs—renal cortex, cortex-medulla boundary, and medulla—and used the gray-level co-occurrence matrix algorithm to extract features from each ROI. A total of 57 features were extracted and processed through an artificial neural network consisting of 58 input parameters, 10 hidden layers, and three output layers (normal, mild and moderate CKD, and severe CKD), with a final classification accuracy of 95.4% (26). Zhang et al. attempted to classify diffuse glomerulopathy using ultrasound radiomics. They extracted a series of 180 ultrasound radiomic features from ultrasound images of patients with IgAN and membranous nephropathy to describe kidney features, reaching the highest accuracy of 0.7647 (27). Similar to these studies, we extracted high-dimensional imaging features from renal ultrasound images and identified 12 most significant independent predictive features. Further, we developed several ML models by combining different classifiers and sequences. Notably, our results showed that the LR classifier performed better than the other two classifiers in the IgAN crescent status classification tasks, with AUC values of 0.838 and 0.810 in the training and test cohorts, respectively.

In this study, although we included numerous baseline clinical data, after the univariate and multivariate logistic regression analyses, only the presence of occult hematuria was an independent predictor in the clinical model. The pathogenesis of crescent formation is related to immune and inflammatory reactions, reflecting the severity of interstitial inflammatory infiltration (10, 28). Crescent formation is due to focal rupture of the glomerular basement membrane (29, 30). Therefore, the presence of occult hematuria reflects the formation of crescents to some extent (31, 32). The clinical**−**radiomic nomogram established by combining the Rad score and the presence of occult hematuria showed sufficient prediction effectiveness in both cohorts. The clinical model reflects the role of baseline clinical information in the noninvasive assessment of the crescent status, while the radiomic model based on ultrasound images involves image quantification. The clinical**−**radiomic model combines the advantages of the clinical and radiomic models, and improves the overall prediction effectiveness of the model.

The present study had some limitations. First, the retrospective design was prone to selection bias. Second, this was a single-center study with a limited sample size; thus, future multicenter studies could provide more generalizable performance verification. Finally, multimodal ultrasound might further improve the accuracy of the established model, which was our future research direction.

In summary, we developed a clinical**−**radiomic nomogram based on clinical and ultrasound radiomic features, which demonstrated high accuracy in differentiating between presence and absence of crescents in IgAN. This nomogram will allow noninvasive assessment of the crescent status in IgAN by providing clinicians with more comprehensive and personalized image information, which is of great significance for the selection of treatment strategies.

## Data availability statement

The raw data supporting the conclusions of this article will be made available by the authors, without undue reservation.

## Ethics statement

The studies involving human participants were reviewed and approved by the First Affiliated Hospital of Anhui Medical University (approval number PJ2022-11-29). Written informed consent for participation was not required for this study in accordance with the national legislation and the institutional requirements. Written informed consent was not obtained from the individual(s) for the publication of any potentially identifiable images or data included in this article.

## Author contributions

Author contributions CZ and XX had full access to all the data in the study and takes responsibility for the integrity of the data and the accuracy of the data analysis. All authors read and approved the final manuscript. Concept and design: CZ, XX and XQ. Acquisition, analysis, or interpretation of data: XQ. Drafting of the manuscript: XQ and LX. Critical revision of the manuscript for important intellectual content: CZ and XX. Statistical analysis: WX and XHM. All authors contributed to the article and approved the submitted version.
